# Difference in virulence and composition of a cariogenic biofilm according to substratum direction

**DOI:** 10.1038/s41598-018-24626-2

**Published:** 2018-04-19

**Authors:** Minh-Huy Dang, Ji-Eun Jung, Hyeon-Mi Choi, Jae-Gyu Jeon

**Affiliations:** 1 Department of Preventive Dentistry, School of Dentistry, Institute of Oral Bioscience and BK 21 plus program, Chonbuk National University, Jeonju, 561-756 Republic of Korea; 2 Department of Dentistry, Presbyterian Medical Center, Jeonju, Republic of Korea

## Abstract

The aim of this study was to investigate the difference in composition and virulence of *Streptococcus mutans* biofilms according to substratum direction. *S. mutans* biofilms (46-h-old) were formed on three different saliva-coated hydroxyapatite (sHA) disc direction groups: downward (discs placed in the direction of gravity), vertical (discs placed parallel to gravity direction), and upward (discs placed opposite to gravity). The 46-h-old biofilms on sHA discs in the upward direction showed the highest biofilm accumulation, colony forming unit (CFU) count, and extracellular polysaccharide (EPS) amount, followed by those in the vertical and downward directions. In the confocal laser scanning microscopy (CLSM) study, the biofilms in the upward direction also showed the highest bacterial count (live or dead cells) and EPS biovolume. Scanning electron microscopy (SEM) analysis confirmed the microbiological and biochemical results. In addition, biofilm density and acid production were higher in the upward direction than those in the other directions. Our findings suggest that substratum direction, which might be related to gravity, strongly influences the formation and virulence of cariogenic biofilms and subsequent initiation of dental caries. Collectively, the differences in the formation and virulence of cariogenic biofilms are related to the direction of tooth surface (occlusal surfaces of mandibular teeth > proximal surfaces > occlusal surfaces of maxillary teeth).

## Introduction

Dental caries is a biofilm-related oral disease that continues to afflict the majority of the world’s population^1^. Recently, the prevalence of the disease has declined due to an improvement of caries prevention strategies such as restriction of sugar consumption and the widespread use of fluoride products. However, dental caries still remains an important health problem, and the occurrence of the disease is closely associated with tooth type and surface^2,3^. Numerous epidemiological studies have shown that occlusal surfaces of posterior teeth are the most susceptible to dental caries^4,5^. The high incidence of dental caries on occlusal surfaces has been related to their narrow and inaccessible surface pits and fissures^6^, indicating that substratum surface morphology is an important factor in cariogenic biofilm (clinically dental plaque) formation and subsequent dental caries initiation.

In addition, a previous study reported that the incidence of dental caries on occlusal surfaces of mandibular posterior teeth was higher than in maxillary posterior teeth^7^. Furthermore, a recent study demonstrated that substratum placed in a vertical position showed less biofilm formation than that in a horizontal position^8^. These findings suggest that cariogenic biofilm formation and subsequent dental caries initiation might be closely related to the direction of tooth surface (substratum). However, despite recent in-depth studies on cariogenic biofilms and dental caries occurrence, few studies have demonstrated differences in cariogenic biofilm formation according to tooth surface direction.

When cariogenic biofilms are sustained on tooth surfaces and exposed to dietary sugars, cariogenic bacteria decrease the pH of the biofilms via glycolysis^9^. Of the bacteria in the biofilms, *Streptococcus mutans* has been regarded as a primary etiologic agent of dental caries. This bacterium efficiently utilizes sucrose to create acidic environments, which can facilitate the growth of aciduric bacteria that lead to dissolution of the tooth enamel and extra-cellular polysaccharide (EPS) synthesis via glucosyltransferases^10^. The EPSs contribute to the structural integrity and stability of a biofilm^11^. However, little has been reported on the influence of the direction of tooth surface on virulence (EPSs and acidogenicity) and viability of *S. mutans* biofilms.

In the present study, we hypothesized that the formation of *S. mutans* biofilms is dependent on the direction of substratum surfaces and consequently cariogenicity of the biofilms will change according to the direction of substratum surface. Accordingly, the aim of this study was to investigate the differences in virulence (EPSs and acidogenicity) and viability of cariogenic biofilms according to the direction of substratum surface using an *S. mutans* biofilm model.

## Results

### Difference in biofilm formation

**Figure 1:**
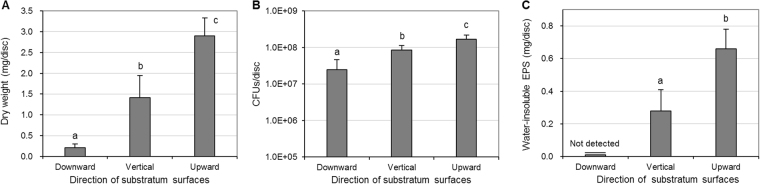
Dry weight (**A**), CFUs (**B**), and water-insoluble EPSs (**C**) of 46-h-old *S. mutans* biofilms formed on sHA discs placed in downward, vertical, and upward directions. Values followed by the same superscript are not significantly different from each other (*p* > 0.05).

*S. mutans* 46-h-old biofilm formation was strongly influenced by the direction of substratum surface. As shown in Fig. 1A,B, and C, the dry weight, colony forming unit (CFU) counts, and amount of water-insoluble EPSs of the 46-h-old biofilms formed on the sHA discs placed in the upward direction were significantly higher than those placed in the vertical and downward directions (p < 0.05). Of the biofilms analyzed, those formed in the downward direction showed the lowest values (p < 0.05). Specifically, the amount of water-insoluble EPSs of the biofilms formed in the downward direction was not detectable via biochemical analysis in the present study (Fig. 1C).

### Difference in acid production

**Figure 2:**
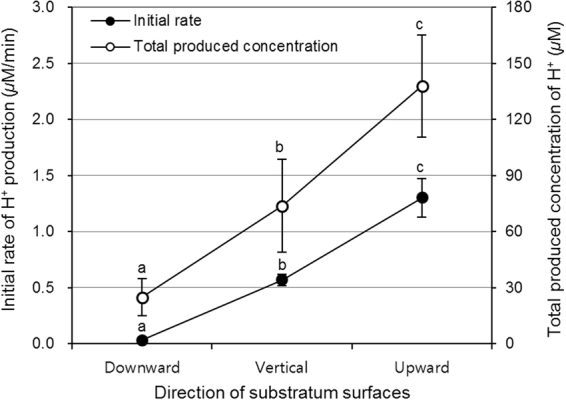
Acid production in 46-h-old *S. mutans* biofilms formed on sHA discs placed in downward, vertical, and upward directions. Changes in initial rate of H^+^ production (0–20 min) and total produced concentration of H^+^ (180 min) in 46-h-old *S. mutans* biofilms, calculated from biofilm pH drop assay data. Values followed by the same superscript are not significantly different from each other (*p* > 0.05).

Acid production in the 46-h-old *S. mutans* biofilms was also influenced by the direction of the substratum surface. Of the 46-h-old biofilms analyzed, biofilms that formed on the sHA discs placed in the upward direction showed the highest initial rate of H^+^ production and total produced concentration of H^+^, followed by those in the vertical direction (p < 0.05) (Fig. 2). Biofilms formed in the downward direction showed the lowest values (p < 0.05).

### Bacterial biovolume and biofilm thickness

**Figure 3:**
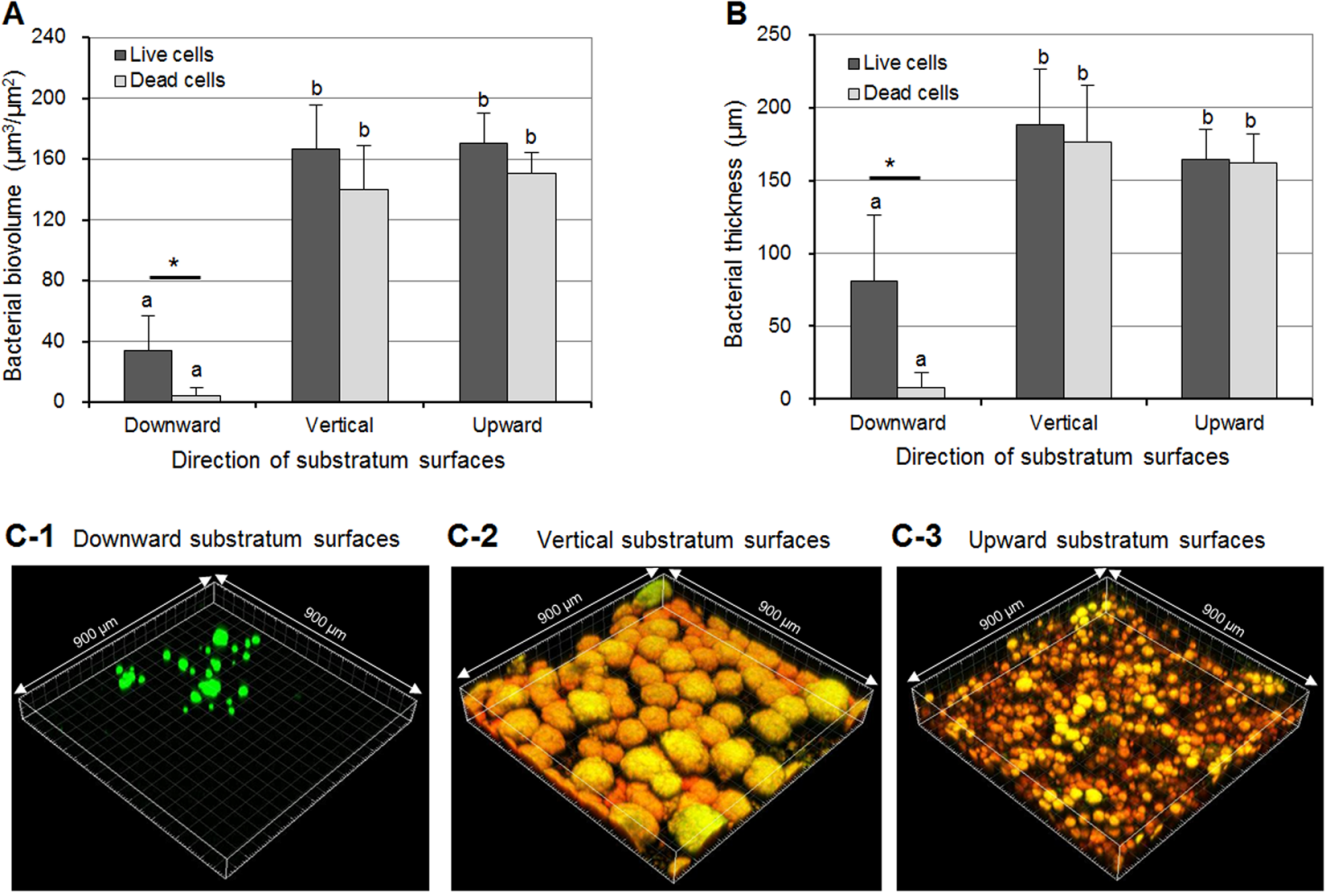
Effect of substratum direction on live and dead cells. (**A**) Bacterial biovolume, (**B**) bacterial thickness of live and dead cells in 46-h-old *S. mutans* biofilms, and (**C**) representative 3-D images of live (green) and dead (red) cells in the biofilms: (C-1) downward substratum surfaces, (C-2) vertical substratum surfaces, and (C-3) upward substratum surfaces. Values followed by the same superscript are not significantly different from each other (*p* > 0.05). *p < 0.05: significantly different from each other.

CLSM was performed to confirm the microbiological and biochemical study results. As shown in Fig. 3, the biovolume of live or dead cells in the 46-h-old *S. mutans* biofilms that formed on the sHA discs placed in the downward direction was significantly lower than those placed in the vertical and upward directions (p < 0.05) (Fig. 3A,C). There was no significant difference in biovolume between the biofilms formed in the vertical and downward directions. However, the biovolume of dead cells in the downward direction biofilms was significantly lower than that of live cells (p < 0.05) compared to the other test groups. The difference in bacterial thickness showed a similar tendency to that of bacterial biovolume (Fig. 3B,C). Figure 3C shows representative bacterial images from the CLSM study, in which the bacterial micro-colonies of the vertical direction biofilms were bigger and more aggregated than those in the upward and downward directions.

### EPS biovolume and biofilm thickness

**Figure 4:**
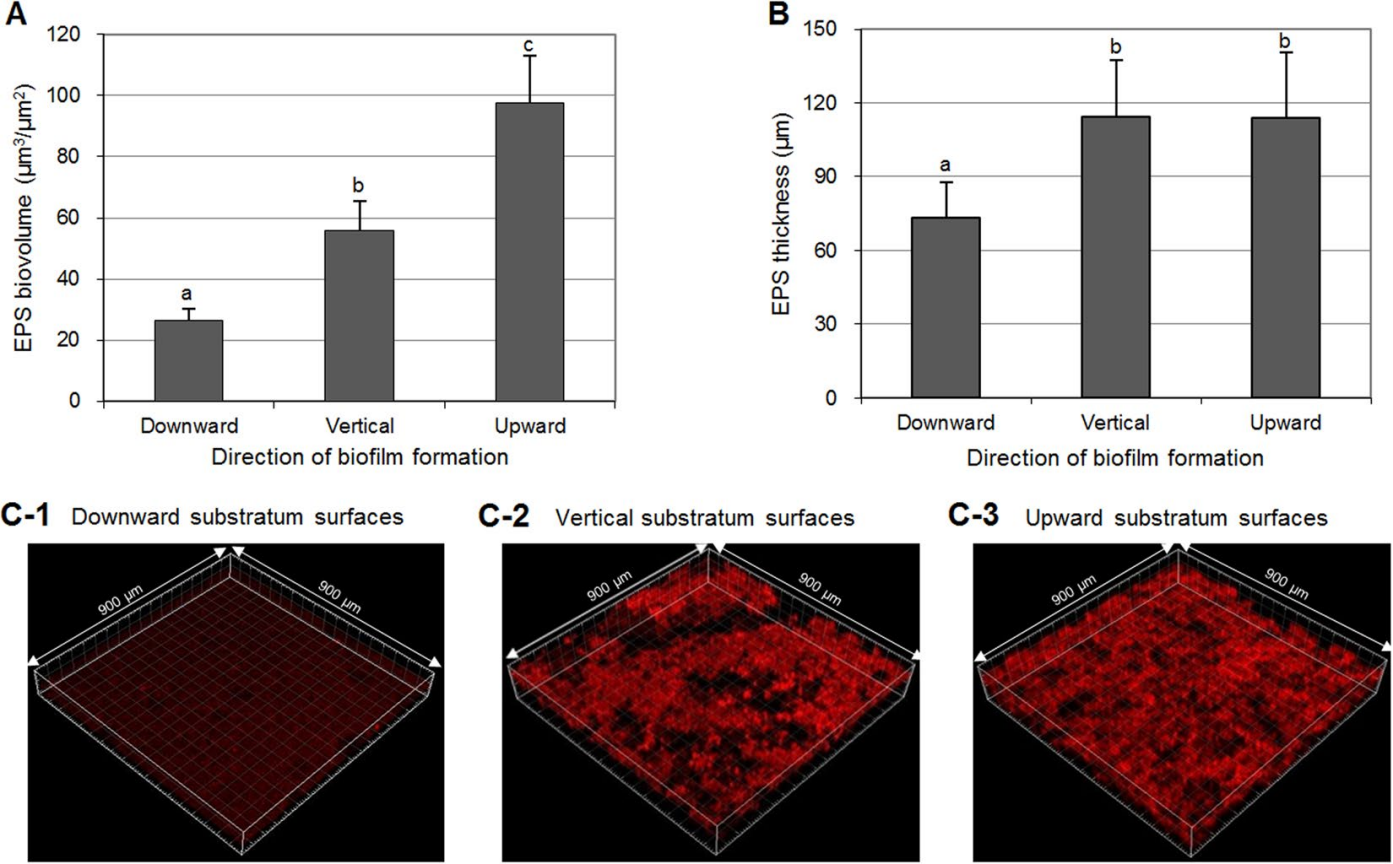
Effect of substratum direction on EPS. (**A**) EPS biovolume, (**B**) EPS thickness of 46-h-old *S. mutans* biofilm, and (**C**) representative 3-D images of EPSs (red) in the biofilms: (C-1) downward substratum surfaces, (C-2) vertical substratum surfaces, and (C-3) upward substratum surfaces. Values followed by the same superscript are not significantly different from each other (*p* > 0.05).

The biovolume and thickness of EPSs in the 46-h-old *S. mutans* biofilms were also influenced by the substratum surface direction. Of the biofilms analyzed, the EPSs in biofilms formed on the sHA discs placed in the upward direction showed the highest values, followed by those in the vertical direction (p < 0.05) (Fig. 4A). However, there was no significant difference between the thickness of EPSs in the upward and vertical directions (Fig. 4B). Generally, the biovolume and thickness of EPSs in the downward direction were the lowest among those in the test groups (p < 0.05). Figure 4C shows representative EPS images from the CLSM study in which the substratum surface direction influenced the biovolume and thickness of EPSs in *S. mutans* biofilms.

### Difference in biofilm density

**Figure 5:**
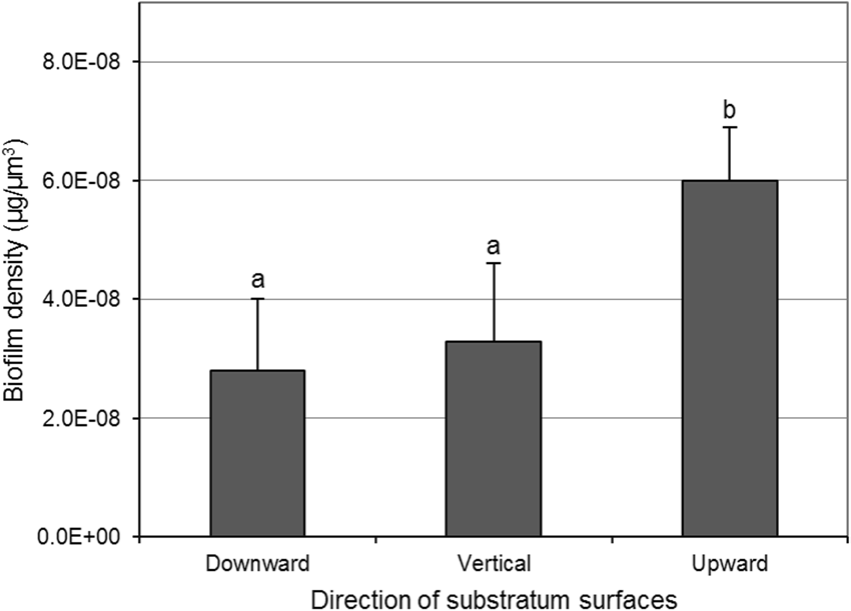
Biofilm density of 46-h-old *S. mutans* biofilms formed on sHA discs placed in downward, vertical, and upward directions. Values followed by the same superscripts are not significantly different from each other (*p* > 0.05).

The density of *S. mutans* biofilms was calculated to investigate the difference in biofilm compactness according to direction of substratum surface. As shown in Fig. 5, the density of 46-h-old *S. mutans* biofilms that formed on the sHA discs placed in the upward direction was higher than those in the vertical and downward directions (p < 0.05). However, there was no significant difference in biofilm density between the biofilms formed in the vertical and downward directions.

### SEM study

**Figure 6:**
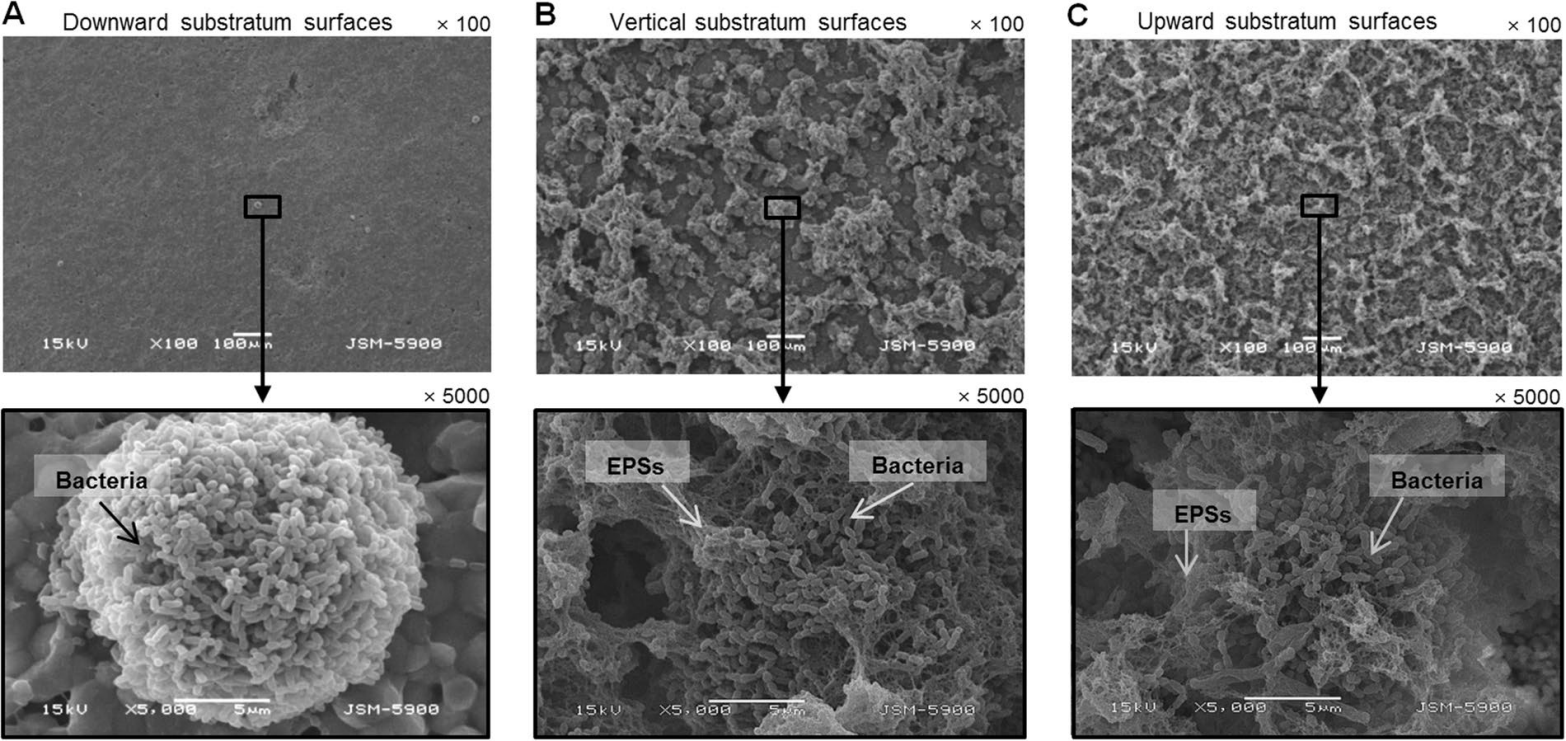
Representative SEM images (×100, ×5000) of 46-h-old *S. mutans* biofilms formed on sHA discs placed in different directions: (**A**) downward substratum surfaces, (**B**) vertical substratum surfaces, and (**C**) upward substratum surfaces.

The SEM images of the 46-h-old biofilms formed on the sHA discs placed in the downward, vertical, and upward directions are shown in Fig. 6. Biofilms that formed in the downward direction showed a few scattered micro-colonies without EPSs. On the other hand, the biofilms that formed in the vertical and upward directions had a number of micro-colonies enclosed by EPSs. The size of micro-colonies in the vertical direction was bigger than those in the downward and upward directions.

## Discussion

In the present study, the direction of substratum surface strongly influenced the biofilm accumulation of *S. mutans* (Fig. 6). Dry weight, which can reflect biofilm accumulation of *S. mutans* biofilms, was highest in the upward direction, followed by those in the vertical and downward directions (Fig. 1A), even though the sHA disc surface area was not different among the three directions. A recent study reported that *Candida albicans* biofilm accumulation is influenced by substratum surface direction^8^. The difference in biofilm accumulation according to substratum surface direction might be related to the force of gravity. Gravity might interfere somewhat with biofilm accumulation on substratum surfaces in the downward and vertical directions and cause the accumulated biofilms to loosen more easily from the surface. On the other hand, gravity might not affect biofilm accumulation on substratum surfaces in the upward direction. According to a previous study, gravity is an essential factor in the accumulation and structure of *S. mutans* biofilms^12^.

Our CFU counts and amount of water-insoluble EPSs in *S. mutans* biofilms support the influence of substratum surface direction in biofilm accumulation. As shown in Fig. 1B,C, the CFU counts and amount of water-insoluble EPSs in *S. mutans* biofilms were the highest in the upward direction, followed by those in the vertical and downward directions. In the downward and vertical directions, gravity might reduce adhesion and accumulation of *S. mutans* cells to the substratum surface. The reduction in biofilm bacterial cells could result in a decrease in EPSs since the synthesis of EPSs depends on the number of *S. mutans* cells. Furthermore, the decrease in EPS amount could facilitate the detachment of biofilm components from the substratum surface in the vertical and downward directions since EPSs contribute to the bulk and physical integrity and stability of biofilms^13^. In general, our data clearly show that the direction of substratum surface strongly influences the accumulation of bacteria and EPSs on surfaces and, consequently, biofilm accumulation.

In the present study, the CFU counts and amount of water-insoluble EPSs in *S. mutans* biofilms in the vertical direction were higher than those in the downward direction (Fig. 1B and C), suggesting that substratum surfaces in the vertical direction are favorable for biofilm formation over those in the downward direction. Although favorable factors in the vertical direction were not identified in the present study, the frictional force resulting from micro-roughness might facilitate *S. mutans* adhesion to the substratum surface and consequently result in an increase in CFU counts and EPS production of biofilms in that direction. Furthermore, the increase in EPSs in the vertical direction could interrupt detachment of dead bacteria from the substratum surface. As shown in results from the CLSM study (Fig. 3A and C), the proportion of dead cell biovolume in the downward direction was lower than that in the vertical direction.

The direction of substratum surface also strongly influenced the shape of bacterial micro-colonies (or embedded in EPSs) of *S. mutans* biofilms. As shown in CLSM and SEM images (Figs 3C and 6), the bacterial micro-colonies (or embedded in EPSs) of biofilms in the vertical direction were bigger and more aggregated than those in the upward and downward directions, suggesting that the direction of substratum surface is an important factor in establishment of micro-colony shape. Furthermore, the change in bacterial micro-colony shape can explain the results in Figs 1B and 3A, which show that biofilms in the vertical and upper directions have a similar biovolume of live cells even though the CFU counts were different. Generally, a more sophisticated study will be needed, as the present study did not investigate the mechanisms of action related to change in micro-colony shape according to substratum surface direction.

Acid production is one of the main virulence factors of *S. mutans* biofilms that leads to the demineralization of the tooth enamel^14^. In the present study, the initial rate of H^+^ production and total produced concentration of H^+^ of *S. mutans* biofilms were highest in the upward direction, followed by those in vertical and downward directions (Fig. 2). This result indicates that the rate and concentration of H^+^ produced by *S. mutans* biofilms clearly depend on the direction of substratum surface on which the biofilms form. The increase in initial rate and total produced concentration in the upward direction might be closely related to the number of viable cells of the biofilms in the direction. As shown in Fig. 1B, the viable cell counts of *S. mutans* biofilms in the upward direction were higher than those in the other directions, which suggests that biofilms positioned in the upward direction can produce a large amount of acid quickly. In the present study, the acid production ability per bacterial colony was not different in all the directions studied (data not shown).

In the CLSM study, the thicknesses of *S. mutans* biofilms (bacteria or EPSs) in the vertical and upward direction were similar to each other, although the dry weight in the upward direction was higher than that in vertical direction (Figs 1A and 3A). This result suggests a difference in density of the biofilms according to direction of substratum surface. Thus, the density of *S. mutans* biofilms in upward, vertical, and downward directions was calculated. As shown in Fig. 5, the density of *S. mutans* biofilms in the upward direction was higher than in the downward and vertical directions. This result indicates that the density of *S. mutans* biofilms depends on the direction of gravity, and that biofilms in the upward direction have a more compact structure than those with downward and vertical directions, which were confirmed by SEM study (Fig. 6). However, the density values of *S. mutans* biofilms in the downward and vertical directions were not different from each other (Fig. 5). This result suggests that there is little variation in influence of gravity on the density of biofilms formed in the vertical and downward directions.

In the present study, our data clearly showed that CFU counts, EPS amounts, biofilm density, and acid production of *S. mutans* biofilms in the upward direction are higher than those in the other direction (Figs 1, 2, and 5). These findings suggest that cariogenic ability of *S. mutans* biofilms formed on occlusal surfaces of mandibular teeth may be stronger than that formed on proximal surfaces or occlusal surfaces of maxillary teeth, even under the same environmental conditions. This may be mainly due to large amounts of acid production by *S. mutans* and highly structured EPS of the biofilms. As reported in a previous study^15^, the presence of highly structured and enlarged EPS-enmeshed micro-colonies could retain produced acids more effectively than those from non-structured bacterial accumulation. In general, our data may explain the results of previous epidemiological studies that show the incidence of dental caries on occlusal surfaces of mandibular posterior teeth is higher than of maxillary posterior teeth^7,16^. Collectively, despite the same area of exposure to *S. mutans*, biofilm accumulation, CFU counts, and EPS amount on substratum surfaces (sHA discs) were highest in the upward direction, followed by those in the vertical and downward directions, which might be related to gravity. Furthermore, biofilms on substratum surfaces in the upward direction showed the highest H^+^ concentration and biofilm density among the direction tested. This finding suggests that differences in the formation and virulence of cariogenic biofilms and subsequent initiation of dental caries are related to the direction of tooth surface (occlusal surfaces of mandibular teeth > proximal surfaces > occlusal surfaces of maxillary teeth).

## Methods

### Direction of substratum surface

**Figure 7:**
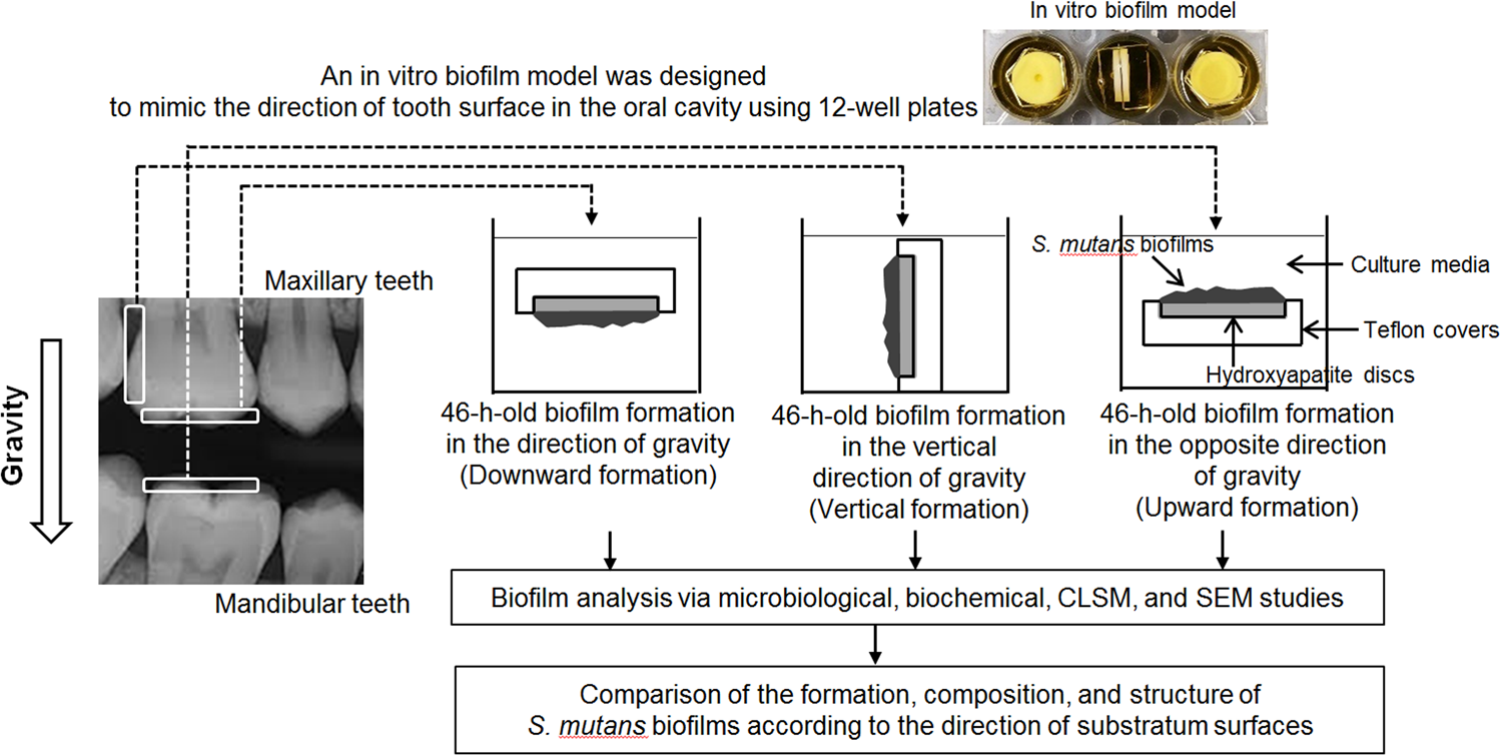
*S. mutans* biofilm formation on saliva-coated hydroxyapatite (sHA) discs placed in different directions and the experimental plan.

Hydroxyapatite discs (HA discs, 2.93 cm^2^; Clarkson Chromatography Products, Inc., South Williamsport, PA, USA) were situated in Teflon covers that allow bacterial cells to be exposed to one-side of the HA disc surface (Fig. 7). The HA discs in Teflon covers were placed in modified orthodontic wire wrapping and divided into three experimental groups depending on HA disc direction: (a) downward group, HA discs placed horizontally in a downward position, allowing biofilms to grow downward; (b) vertical group, HA discs placed in a vertical position, allowing biofilms to grow laterally; and (c) upward group, HA discs placed horizontally in an upward position, allowing biofilms to grow upward.

### Biofilm formation and experimental scheme

The study was approved by the Institutional Review Board (IRB) of Chonbuk National University (IRB File no.: JBNU 2017-02-007), and written informed consent was obtained from all participants. The methods in the present study were performed in accordance with the approved guidelines, and experimental protocols were approved by the IRB of Chonbuk National University. *S. mutans* UA159 (ATCC 700610; serotype c) biofilms were formed on saliva-coated HA (sHA) discs. The sHA discs were generated by incubation with filter-sterilized (0.22 μm low protein binding filter) human whole saliva for 1 h at 37 °C. The *in vitro* biofilm model was designed to mimic the direction of tooth surface in oral cavity (Fig. 7). For biofilm formation, the sHA discs were situated in Teflon covers and transferred to a 12-well plate (placed in a vertical, downward, or upward position) containing 1% sucrose UTE broth with 2–5 × 10^6^ CFU/ml (6 ml/disc) *S. mutans* UA159. The biofilms were grown undisturbed for 22 h to allow initial biofilm growth. From this point, the culture medium was changed two times (at 22 and 31 h) until the biofilm was 46 h old. In the present study, biofilms of 46 h old were defined as mature biofilms^17^. The 46-h-old biofilms were then analyzed via microbiological and biochemical methods, confocal laser scanning microscopy (CLSM), and scanning electron microscopy (SEM) (Fig. 7) to determine the difference in virulence (EPSs and acidogenicity) and viability of cariogenic biofilms according to substratum surface direction.

### Biofilm formation analysis

For biofilm formation analysis, the sHA discs containing 46-h-old *S. mutans* biofilms were released from Teflon covers. The discs were then transferred into 2 ml of 0.89% NaCl and sonicated in an ultrasonic bath for 10 min to disperse the biofilms. The suspension was homogenized by sonication at 7 W for 30 s after adding 3 ml of 0.89% NaCl. An aliquot (100 μl) of the homogenized suspension was serially diluted, plated on BHI agar plates, and incubated to determine viable bacterial counts. To determine the biofilm dry weight, the remaining solution (4.9 ml) was centrifuged (3,000 × g) for 20 min at 4 °C. The biofilm pellet was resuspended and washed twice in the same volume of water. The washed pellet was then lyophilized and weighed as detailed elsewhere^18^. The amount of water-insoluble EPSs was then extracted from the dry pellet using 1.0 N NaOH and ethanol precipitation before assessing by the phenol-sulfuric acid method with glucose as a standard^19^.

### Acid production analysis

Acid production in the 46-h-old *S. mutans* biofilms was determined using the glycolytic pH drop assay as described elsewhere^20^. Briefly, the *S. mutans* biofilms were incubated in 20 mM potassium phosphate buffer (pH 7.2) for 1 h to deplete endogenous catabolites and transferred to a salt solution (50 mM KCl + 1 mM MgCl_2_, pH 7.0). The pH was adjusted to 7.2 with 0.2 M KOH solution. Glucose was then added to a final concentration of 1% (w/v). The decrease in pH was assessed using a glass electrode over a period of 180 min (Futura Micro Combination pH electrode, 5 mm diameter; Beckman Coulter Inc., CA, USA). The acidogenicity of the biofilms was evaluated using the initial rate of H^+^ production and the total produced concentration of H^+^ calculated by pH values of pH drop curves. The initial rate of H^+^ production (y) was derived from the equation: y = (H^+^ concentration at 20 min – H^+^ concentration at 0 min)/20. The total produced concentration of H^+^ (y) was derived from the equation: y = H^+^ concentration at 180 min – H^+^ concentration at 0 min.

### Live and dead bacterial cell staining

The 46-h-old biofilms were stained at room temperature in the dark for 30 min using a FilmTracer LIVE/DEAD Biofilm viability kit L10316 (Invitrogen, Molecular Probes Inc., Eugene, OR, USA). The final concentrations of SYTO9 and propidium iodide (PI) were 6.0 μM and 30 μM, respectively. The excitation/emission wavelengths were 480/500 nm for SYTO 9 and 490/635 nm for PI for collecting the fluorescence. The stained live and dead bacterial cells were observed with an LSM 510 META microscope (Carl Zeiss, Jena, Germany) equipped with argon-ion and helium–neon lasers. All confocal fluorescence images were taken with an EC Plan-Neofluar 10x/0.30 M27 objective lens. Three independent experiments were performed, and five image stacks per experiment were collected (*n* = 15). A stack of slices in 6.4 μm step sizes was captured from the top to the bottom of the biofilm. The biovolume and thickness of live and dead cells were quantified from the entire stack using COMSTAT image-processing software^21^. The biovolume is defined as the volume of the biomass (μm^3^) divided by the substratum (hydroxyapatite surface) area (μm^2^). The three-dimensional architecture of the biofilms was visualized using Imaris 8.0.2 (Bitplane, Zurich, Switzerland). The original confocal data was uploaded to Imaris 8.0.2 software and the intensity of green and red fluorescence in full thickness of biofilm layers were captured automatically. The software reconstructed the 2-dimentional intensity of fluorescence in all the layers to a 3-dimentional volume stack^22^.

### EPS staining

The EPSs of 46-h-old biofilms were also investigated by simultaneous *in situ* labeling as described elsewhere^23^. Briefly, Alexa Fluor^®^ 647-labeled dextran conjugate (1 μM, 10,000 MW; absorbance/fluorescence emission maxima 647/668 nm; Molecular Probes Inc., Eugene, OR, USA) was added to the culture medium during the formation of *S. mutans* biofilms (at 0, 22, and 31 h) to label the newly formed EPSs. As described above, the stained EPSs were observed with an LSM 510 META microscope (Carl Zeiss, Jena, Germany) (objective: EC Plan Neofluar 10x/0.30 M27) equipped with argon-ion and helium–neon lasers and visualized using Imaris 8.0.2. A stack of slices in 7.8 μm step sizes was captured from the top to the bottom of the biofilm. Three independent experiments were performed, and five image stacks per experiment were collected. The EPS biovolume and thickness were quantified from the confocal stacks using COMSTAT.

### Biofilm density

Density of the 46-h-old biofilms was calculated using the dry weight, which was derived from the biochemical study above, and the total biovolume of the biofilms (live cells + dead cells + EPSs), which was derived from the CLSM study above. The biofilm density (μg/μm^3^) is defined as the dry weight (μg/μm^2^) divided by the total biovolume of the biofilm (μm^3^/μm^2^).

### Scanning electron microscopy (SEM)

For SEM analysis, the 46-h-old biofilms were washed three times with 0.1 M cacodylate buffer and fixed with a 3% glutaraldehyde solution for 1 h, followed by post-fixation with a 1% osmium tetroxide solution for 1 h^24^. The biofilms were dehydrated using a graded ethanol series (30–100%) and penetrated with nitrogen gas. Finally, the biofilm samples were sputter-coated with gold–palladium and observed by SEM (JSM-5900, Jeol, Japan).

### Statistical analysis

All experiments (except CLSM and SEM) were performed in duplicate, and at least four different experiments were conducted. The data are presented as mean ± standard deviation. Intergroup differences were estimated using one-way analysis of variance, followed by a post hoc multiple comparison (Tukey) test to compare multiple means. Values were considered statistically significant when the p value was <0.05.
